# Scientific Evidence about the Risks of Micro and Nanoplastics (MNPLs) to Human Health and Their Exposure Routes through the Environment

**DOI:** 10.3390/toxics10060308

**Published:** 2022-06-08

**Authors:** Ana Clara Bastos Rodrigues, Gabriel Pereira de Jesus, Dunia Waked, Gabriel Leandro Gomes, Thamires Moraes Silva, Victor Yuji Yariwake, Mariane Paula da Silva, Antônio José Magaldi, Mariana Matera Veras

**Affiliations:** 1 Laboratory of Experimental and Environmental Pathology–LIM05, Department of Pathology, Faculty of Medicine, University of Sao Paulo, Sao Paulo 01246-000, Brazil; anaclarabr@usp.br (A.C.B.R.); gabrielamaral5555@gmail.com (G.P.d.J.); duniawaked@usp.br (D.W.); gomesglbio@gmail.com (G.L.G.); moraesthami44@gmail.com (T.M.S.); victoryuji.13@gmail.com (V.Y.Y.); marianepaula@ymail.com (M.P.d.S.); 2 Kidney Research Laboratory–LIM12, Department of Nephrology, Faculty of Medicine, University of Sao Paulo, Sao Paulo 01246-000, Brazil; biomag@usp.br

**Keywords:** microplastics, ecosystem, human health, toxicity

## Abstract

Nowadays, a large amount and variety of plastic is being produced and consumed by human beings on an enormous scale. Microplastics and nanoplastics (MNPLs) have become ubiquitous since they can be found in many ecosystem components. Plastic particles can be found in soil, water, and air. The routes of human exposure are numerous, mainly involving ingestion and inhalation. Once ingested, these particles interact with the gastrointestinal tract and digestive fluids. They can adsorb substances such as additives, heavy metals, proteins, or even microorganisms on their surface, which can cause toxicity. During inhalation, they can be inhaled according to their respective sizes. Studies have reported that exposure to MNPLs can cause damage to the respiratory tract, creating problems such as bronchitis, asthma, fibrosis, and pneumothorax. The reports of boards and committees indicate that there is little data published and available on the toxicity of MNPLs as well as the exposure levels in humans. Despite the well-established concept of MNPLs, their characteristics, and presence in the environment, little is known about their real effects on human health and the environment.

## 1. Introduction

Nowadays, a wide variety and large quantity of plastic is being produced and consumed on a large scale by human beings. Plastics are synthetic polymers mainly derived from the petrochemical industry. Although some sources of biodegradable plastics are produced by natural sources (cellulose, cornstarch, soybeans, etc.), those coming from the oil industry are the most commonly manufactured and can cause a greater impact on nature since they remain longer in the environment [1].

Since it is mostly a synthetic polymerized product, plastic has desirable characteristics when considering durability, resistance, inertia, and its low cost. These properties, while beneficial to industry, have created a significant problem for the environment and human health. The pollution of the environment and the oceans with large amounts of plastic, in all its varieties, has become a global issue in environmental pollution [2].

As previously mentioned, synthetic plastic has incredible characteristics for use in industry, but these same characteristics make these products virtually indestructible due to their composition and material hardness. When in contact with air, soil, and fresh or saltwater, plastic deteriorates into smaller particles called micro and nanoplastics (MNPLs). Consequently, long and short fragments are released from the chains of polymerized organic molecules which can remain in the environment for hundreds of years [3].

Microplastics (MPs) are plastic particles that range from 1 μm to less than 5 mm, while nanoplastics (NPs) are plastic particles smaller than 1 μm [4]. MNPLs are mainly classified in two ways—primary or secondary—taking into account their origin [5]. Primary MNPLs are plastic particles intentionally manufactured to have a small size (1 μm to less than 5 mm), e.g., pellet beads, which are used as raw material for the production of cosmetics, such as toothpastes, exfoliating treatments, body wash, and other personal care products.

Secondary MNPLs are products derived from the degradation and fragmentation of larger plastics, such as bottles, tire and road wear particles (TRWPs), or caps which generate plastic microparticles. This fragmentation occurs due to mechanical actions, UV radiation, temperature, humidity, etc., producing both micro and nanoplastics [6]. In addition to these two types of MNPLs, synthetic microfibers used in the manufacture of clothing and fabrics are also found in the environment [7]. These filaments are released during processes such as washing, reaching urban sewers, and consequently, the environment, and affecting human health [8].

Despite the well-established concept of microplastics and nanoplastics (MNPLs) and their characteristics and presence in the environment, little is known about their real effects on human health. This review considers the scientific evidence of their harmful effects on the environment and human health.

## 2. Routes of Exposure

**Figure 1:**
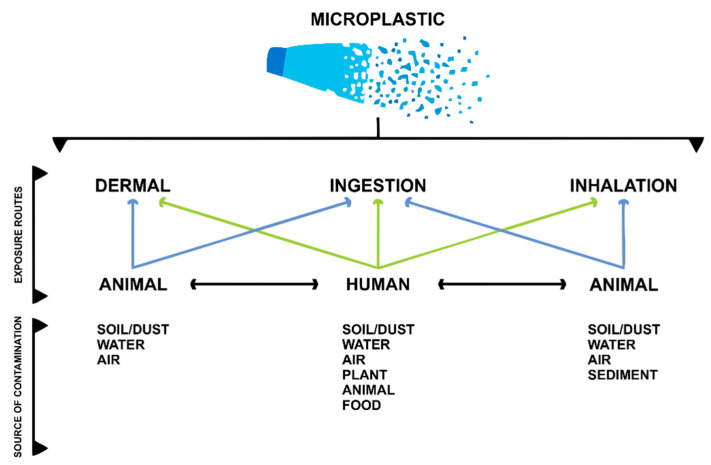
Exposure routes for microplastics in the environment. Modified from Enyoh et al., 2020 [9].

Micro and nanoplastics (MNPLs) have become ubiquitous since they can be found in many ecosystem components. Plastic particles can be found in soil, water, and air. There are many routes of human exposure to microplastics. These can include oral exposure through contaminated water and food (mainly of marine origin); via the dermal route through the use of soaps, scrubs, or via contact with soil; and via inhalation through the precipitation of particles in the air [9], which was found in pulmonary tissue samples from the first study to identify MNPLs in lung tissues [10]. Figure 1 shows a summary of the main exposure routes to humans and animals, showing that humans can experience more exposure to to MNPLs from these different sources.

### 2.1. Respiratory Exposure to Micro and Nanoplastics

MNPL particles, derived from the degradation of plastic in the environment, have been observed in atmospheric precipitations. Depending on the sizes of these particles, they can be inhaled. We must clarify that particles capable of entering through the nose and mouth and being deposited in the upper respiratory tract are classified as inhalables, and particles that are deposited in the lungs are classified as breathable [11]. These particles may be subject to non-specific host defense mechanisms that remove mucus through the mucociliary process. Alveolar macrophages may capture these particles and transport them to the intestine to be removed [12].

Studies have reported that exposure to MNPLs can cause damage to human health, creating problems such as bronchitis, asthma, fibrosis, and pneumothorax. In an experimental study with rats, in which the toxicological potential of MNPLs inhaled for 14 days was investigated, it was observed that levels of transforming growth factor beta (TGF-beta), factors related to fibrosis, and tumor necrosis factor alpha (TNF-alpha) were altered [13].

Studies with polystyrene nanoplastics using alveolar basal epithelial cells (A549) have shown that they are able to disturb gene expression, resulting in inflammatory responses and the launch of apoptosis pathways, particularly when smaller PS-NPs are used. The results of one study suggest that nanoplastics can cause definite damage and functional disturbance to human and mammalian respiratory systems [14].

Regarding the involvement of MNPLs in the respiratory tract of humans, for the first time, a recent study identified plastic particles ranging in size from 1.60 to 5.58 μm in the bronchoalveolar region in more than 50% of analyzed lung samples, confirming that the respiratory system is an important route of exposure and that the lungs act as a site of accumulation of MNPLs in human beings [10]. Environmental exposure to MNPLs through the air occurs through several sources, such as synthetic textiles, tire erosion, synthetic rubber, and urban dust [15]. Other sources of airborne MNPLs include plastics from clothes and household furniture. Of note are synthetic textiles, which may be responsible for human exposure in both internal and external environments [16].

The persistence of these particles in the atmosphere is determined by atmospheric precipitation, which is influenced by rain, wind, local conditions, and particle size. Particles with lower densities can be easily carried by the wind, which causes the contamination of terrestrial and aquatic environments [17,18].

Plastic nanoparticles have diameters smaller than 1 μm. These nanoparticles can generate distinct toxicities due to their smaller dimensions [19,20]. The inhalation of MPs and NPs is related to some pathologies and a higher incidence of cancer. Studies carried out using animal models indicate that exposure can induce granulomatous lesions [21,22,23]. MNPLs can have the same toxicity as other atmospheric nanoparticles, which makes it difficult to compare them. Therefore, it is necessary to carry out studies regarding the toxicity of different sizes of polymers and their surface properties [24].

With the arrival of the new coronavirus, the use of masks has become essential for the population, with N95 surgical masks being the most effective at reducing the risk of virus transmission. However, due to the scarcity of masks, people have chosen to use masks made of other materials, such as cotton, nylon, clothing cloth, and textile mixed with polypropylene. During the process of disinfecting masks, the fabric may suffer wear and tear [25,26], causing distress in the material, leading to the risk of inhalation through respiration [27,28].

Li and colleagues [29] carried out a study using seven types of masks—N95, cotton, sky surgery, ply surgery, fashion, activated carbon, and non-woven—for the detection of MNPLs using Raman spectroscopy and infrared spectrometry. The N95 mask performed well and reduced particle inhalation even after disinfection. In addition, fashionable cotton masks and those made of non-woven fabrics and activated carbon were shown to reduce the risk of MNPL inhalation. When compared to not wearing a mask, it was concluded that using the above-mentioned masks for at least two hours could reduce the inhalation of plastic particles [29].

### 2.2. Oral Exposure to Micro and Nanoplastics

The presence of MNPLs in food and beverages has evidently increased. Recent studies have reported the detection of plastic particles in common food products, such as salt, milk, honey, fruits, vegetables, mineral water, and marine foods. Thus, human exposure through ingestion is quite likely and needs to be studied while taking into account the entire food production chain, from cultivation to consumption [30,31,32].

Despite being a subject of interest, there are still many obstacles in relation to the methodologies used to detect MNPLs in foods and beverages and characterization parameters, such as exposure dose. A first issue is the diversity of these particles, which can have different chemical compositions, in addition to different densities, sizes, and formats, which makes it difficult to standardize techniques. In addition, there is an absence of reference values and precise definitions [32]. Such obstacles make research on oral exposure to MNPLs challenging. However, advances have been reported. Recent studies estimate that each person ingests an amount of MNPLs ranging from 39,000 to 52,000 particles annually [31]. Such findings highlight the importance of and the need for further investigation on this subject [33,34].

Some of the most studied foods are those of aquatic origin [35]. Generally, fish accumulate MNPLs in their gills, liver, and intestine, parts that are generally not eaten by humans [36]. However, in some filtering animals, such as shrimps, a relatively large amount of MNPLs was observed. It is estimated that consumers of these animals ingest about 11,000 particles annually [37]. In addition to food of aquatic origin, commercial mineral water showed traces of plastic particles, even in bottled water in glass containers [38], while polypropylene particles have been identified in various brands of table salt [39]. Plastic particles have also been found in fruits, such as apples and pears, and vegetables, such as potatoes, broccoli, carrots, and lettuce [40]. The presence of microplastics in fecal samples from humans reinforces the idea that particles are ingested by humans [41]. However, the effects of this kind of oral exposure are still unclear.

**Figure 2:**
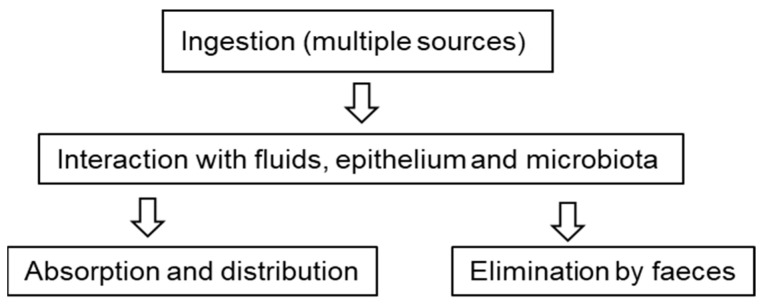
Oral exposure to microplastics. Possible routes and interactions that are currently under study.

Once ingested, particles can interact with the gastrointestinal tract and digestive fluids. Despite being considered chemically inert particles, plastics can adsorb substances, such as additives, heavy metals, proteins, or even microorganisms, on their surface, which can cause greater toxicity. In this situation, MNPLs work like a Trojan horse, bringing a series of environmental contaminants with them. When ingested, the particles can interact with the mucus that lines the gastrointestinal tract, with the epithelial cells themselves, and even with intestinal microbiota, causing cellular responses and diverse physiological changes (Figure 2) [42,43,44,45].

Recent research suggests that, depending on the size, particles can be internalized by intestinal epithelial cells (endocytosis) or they can pass between intestinal cells (paracellular transport) [46]. Animal studies have shown the distribution and deposition of MNPLs in organs such as the kidneys, liver, and lymph nodes [47,48,49]. However, this systemic distribution remains controversial, and further studies are needed to corroborate this hypothesis.

### 2.3. Dermal Exposure to Micro and Nanoplastics

Although oral exposure is the most notable type of exposure, there are also other types of exposure—one of these is dermal exposure. Even though it is a less efficient route, studies show that micro and nanoplastics can cross the dermal barrier [50]. These nanoparticles are applied, for example, in cosmetics and in the continuous reduction of textile microfibers. In addition, microplastic microspheres (less than 1 mm in diameter) are widely used in dermal exfoliation products, such as toothpastes and denture restorations [51].

Another area in which dermal exposure is discussed is in medicine. In suturing, for example, plastics are known to induce low inflammatory reactions and a foreign body reaction with fibrous encapsulation. In a study of mice, the effects of polyethylene and polyvinyl chloride (PVC) were evaluated, showing that polyethylene was associated with lower inflammation compared to PVC. However, micro and nanoplastics can also induce inflammation and foreign body reactions, with differences in surface properties leading to different results. Human epithelial cells undergo oxidative stress from exposure to micro and nanoplastics, confirming the need for studies that evaluate the effects of exposure to MNPLs [50].

## 3. Epidemiological Studies

Epidemiological studies have already indicated that adverse respiratory and cardiovascular effects are closely linked to air pollution via environmental atmospheric particles [52]. Some studies also demonstrate possible significant correlations between BPA levels (adsorbed in both air and food) in urine and cardiovascular disease and type 2 diabetes [53]. Moreover, recent data have shown the presence of airborne MNPLs in human lung tissue [10] and the bloodstream [54]. Atmospheric research on MNPLs highlights that they can impact remote and under-developed areas that do not have any local sources of plastic [55]. In addition, the dynamics of ocean circulation and marine currents represent important space–time scales in terms of the destination, transport, and effects of micro and nanoplastics on the environment, affecting fauna, and consequently, human life [56].

In addition to the respiratory tract, MNPLs can be ingested by several organisms, mainly by species widely consumed by humans [57,58,59]. However, it is still not fully understood how the interaction between human organic systems and these particles work [60]. This is mainly due to the absence of extensive and significant epidemiological studies to detect their impacts at the population level and the lack of technology available to detect and track these particles in vivo. Research is fundamental for understanding how atmospheric air and the main foods established by nutritional health standards are contributing to the transport of microplastics by the human body, as well as the risks to health [35].

Reports of boards and committees, such as the Scientific Council of the European Commission, Science Advice for Policy by European Academies (SAPEA), and the World Health Organization (WHO), show that there is little data published and available on the toxicity of MNPLs, as well as the exposure levels in humans [60,61]. These reports also highlight some of the main challenges when trying to gather concrete information about the relationships between MNPLs and human health [62].

## 4. Experimental Studies

### 4.1. In Vivo

Since human exposure to MPs and NPs occurs mainly via ingestion, in vivo studies have been prioritized in animal models for a better understanding of the biological effects of these particles on humans. Research indicates that MNPLs, after being adsorbed by the organism, are systemically distributed to organs and tissues, both through the blood and lymphatic currents [63].

According to Stock and colleagues [64], in a study using a murine model and oral exposure via gavage, the histological absence of detectable lesions and inflammatory responses was observed, suggesting that the particles taken in through oral exposure under experimental conditions did not represent relevant acute risks to the health of mammals. In contrast, in another study, a negative load of polystyrene nanoparticles (PS-NPs) in the lungs, testis, spleen, kidney and heart of an animal model after oral administration was observed [49]. These divergent results may be due to the properties of the particles, such as the size, concentration, and dosage.

Questionable findings may also arise, such as the study carried out by Deng and colleagues [65], which identified a high number of 5 µm and 20 µm polystyrene microplastic (PS-MPs) particles crossing the intestinal barrier and distributing to organs, while most studies in mammals have suggested the low oral bioavailability of microplastic particles.

Recent publications have elucidated mechanisms of plastic absorption in female rats and the effect of plastic absorption on the reproductive system, indicating that these polystyrene particles cause fibrosis through the Wnt/B-Catenin signaling pathway and the apoptosis of granular cells in the ovary, resulting in decreased ovarian reserve capacity [66]. In addition, MNPLs also function as adsorbents for heavy metals, causing toxicity in combination with other pollutants in the environment. With respect to testicular toxicity, Hou and colleagues [67] demonstrated a reduction in viable sperm from the epididymis after exposure to 5 μm polystyrene particles in mice, showing atrophy, shedding, and the apoptosis of sperm cells at all levels of the testis after exposure.

With respect to in vivo tracing in aquatic organisms, a study was developed to improve the tracking, transport, and localization of MNPLs in shrimp, medaka, zebrafish, and water fleas, which involved real-time observation for the whole in vivo process of ingestion and egestion in a zebrafish model using manufactured fluorescent fibers. In shrimp, medaka and water fleas, the fibers were observed directly using a fluorescent microscope without dissection [68].

### 4.2. In Vitro

In vitro studies have highlighted important characteristics of MNPLs, such as their hydrophobicity. According to Wright and Kelly [69], the hydrophobicity of these particles is correlated to their more effective transport in intestinal mucus, influencing the adsorption of proteins to the surface of the particle, suggesting that internalization via “M” cells and persorption were the most likely mechanisms responsible for MNPL uptake. In contrast, in vitro studies have also demonstrated that the intestinal epithelium is an important and robust barrier against these types of materials [70].

The size of particles is also an influential factor; for example, 100 nm PS-MNPLs showed greater intestinal toxicity than 5 mm PS-MNPLs. Such studies suggest that direct cytotoxicity assays for PS-MNPLs may erroneously assess their intestinal effects, offering new insights into assessing the toxicity of PS-MNPLs via oral exposure [70].

Another important point relates to in vitro studies using cells from the digestive tract, which is an important target organ with respect to the absorption and distribution of MNPLs [71]. To investigate their effects in relation to the toxicological evaluation of plastic components, some research has been undertaken using differentiated Caco-2 cells, an important experimental model in the study of the intestinal epithelial barrier, especially when combined with HT29 or Raji-B cells [72]. Research has suggested [70] that the digestive process does not alter the chemical constitution of PS-MNPLs, which may be an explanation for the reduced toxicity of MNPLs. In another study, an absence of toxicity was observed in an intestinal cellular model; despite this, it was found that MNPLs could be absorbed by Caco-2 cells [64]. Due to this absorption by the cells, suggesting damage to the plasma membrane, we can infer that MNPLs may induce indirect harmful effects to the gastrointestinal tract, increasing the pro-inflammatory effects of these components.

With respect to acute toxicity in vitro, Hesler et al. [73] evaluated the placental and intestinal barrier using advanced in vitro co-culture models, showing that PS-MNPLs did not have important embryotoxic and genotoxic effects. In recent publications, an increased level of pro-inflammatory cytokines has been demonstrated when plastics are present in high concentrations, consequently causing inflammatory and oxidative lesions. According to previous studies, MNPLs can induce cell death, inflammation, and oxidative stress in various types of cells, decrease cell viability, induce cell apoptosis, alter mitochondrial membrane potential, and deregulate mitochondrial function in lymphocytes, impairing immune function.

As can be seen, size is an important matter—micro and nanoplastics can be ingested by organisms and appear to have some impact on biological functions due to their small size and biological penetration, but nanoplastics seem to have more serious effects. A study on MNPLs (polystyrene) ranging in size from 50 nm, 500 nm, and 5 μm (PS50, PS500, and PS5000), which aimed to analyze the interaction and distribution of these particles with cell membranes, showed that PS particles could enter cells through endocytosis and passive membrane penetration, especially PS50 and PS500, which were both able to cross the cell membrane via energy-independent membrane penetration, though through different pathways. PS50 was endocytosed by RBL-2H3 cells through clathrin-mediated, caveolin-mediated pathways and macropinocytosis, while PS500 was endocytosed via macropinocytosis [74]. PS5000 had no obvious adsorption on the cell because its large particle size made it difficult for it to diffuse to the membrane surface.

## 5. Gaps in Knowledge

Currently, much is being said about MNPLs and their presence in the environment as a pollutant. Despite promising studies, after many years of investigation, little is known about the real toxic and adverse effect of these particles for humans. Furthermore, most of the experimental studies undertaken so far have used nanopolystyrene (MNPLs) as a model for all microplastics. Thus, caution is needed, because we are not sure whether other types of microplastics have similar characteristics and whether they elicit the same responses. Studies have already reported changes in murine models relating to reproductive disorders and inflammatory, respiratory, and intestinal toxicity changes, and have suggested that these particles act in a toxic manner in the body.

In the past 10 years, research has shown that human beings are indeed consuming more and interacting more with plastic microparticles, both through contact, as well as through inhalation and the consumption of food and water. There is great difficulty in biomonitoring MNPLs in the human body, as the existing techniques lack precision [75], making the data obtained unreliable due to their lack of accuracy. Therefore, improvements in the techniques and research methods on animal models are important because the effects of MNPLs on human health are increasingly concerning. To fill the knowledge gaps, it is necessary to ensure more reliable and accurate analysis, standardizing the quantification of MNPLs in the environment and promoting better laboratory standards for research into these particles.

Current exposure scenarios are still speculative and imprecise; however, we suggest that, according to recent studies using animal models, MNPLs may have toxic potential, since they can be absorbed by organs and distributed in human beings.

## Data Availability

Data were obtained through research in virtual libraries, such as Pubmed, Web of Science, and Google Scholar. The review was carried out in a narrative way, without systematic criteria for the search.
